# A review on papilla preservation flap techniques

**DOI:** 10.6026/973206300211463

**Published:** 2025-06-30

**Authors:** Pragya G., Anitha Logaranjani, Prashanthi P., Jaideep Mahendra

**Affiliations:** 1 Department of Periodontics, Meenakshi Ammal Dental College and Hospital, Chennai, Tamil Nadu, India

**Keywords:** Interdental papilla, periodontal flap surgery, papilla preservation flap, esthetics, minimally invasive

## Abstract

Periodontal therapy must necessarily consider esthetic appearance. Therefore, it is important to maintain gingival marginal anatomy with as much as the height of papilla during the course of the periodontal therapy. Surgical therapy used in periodontal defects in maxillary anteriors is possible in an esthetic manner only when integrity of the papilla is preserved. Hence, various flap designs have been developed such as papilla preservation flaps and minimally invasive surgical approaches with papilla elevation or without papilla elevation to reduce the risk of early wound failure. Several surgical techniques are used for primary flap closure and interdental tissue preservation, including various papilla preservation flaps, single flap approaches, soft and connective tissue wall techniques, MIST, M-MIST, and the "Whale's tail" technique.

## Background:

Periodontal therapy primarily aims to halt the progression of periodontal disease and associated damage by regenerating lost periodontal tissues [1]. Surgical intervention targets the elimination of pockets, establishing a stable, easily maintainable state and fostering periodontal regeneration. This involves reconstructing lost periodontal structures, characterized by the formation of cementum, collagen fibers, periodontal ligament and bone [2]. Optimal periodontal therapy should aim to achieve a state of periodontal well-being characterized by the absence of inflammation, periodontal pockets and the patient's ability to sustain both oral health and functionality, along with aesthetics [3]. Maintaining papillary integrity is crucial, especially in treating periodontal defects in the maxillary anterior region, to avoid unsightly gingival architecture resulting from surgery. Preservation of gingival marginal anatomy and papillary height is essential throughout therapy. While non-surgical approaches are often preferred for maxillary anterior dentition, surgical intervention becomes necessary in unavoidable cases, though it risks papillary shrinkage and exposure of interproximal embrasures [4]. Surgical techniques must prioritize papillary preservation to minimize early wound failure risks. Flap designs like papilla preservation flaps aim to maintain the integrity of defect-associated papilla, creating a tunnel-like incision to stabilize blood clots and enhance wound healing [5]. Maximizing gingival tissue preservation ensures complete coverage of regenerative material in osseous defects. Various surgical approaches, including Papilla preservation flap, Modified papilla preservation flap, Simplified papilla preservation flap, Single flap approach, Entire papilla preservation flap, Minimally Invasive Surgical Techniques (MIST), Modified - Minimally Invasive Surgical Techniques (M-MIST) and the "Whale's tail" technique, facilitate primary flap closure and interdental tissue preservation [6]. Therefore, it is of interest to review on papilla preservation flap techniques to enhance periodontal treatment outcomes while prioritizing esthetics and functional restoration.

## Interdental papilla and pink esthetics:

The interdental papilla, once viewed simply as a barrier against food debris, serves a vital role in protecting periodontal structures from oral environment. Preservation of papillary integrity is crucial in dental treatment, as traditional surgical methods can flatten or damage it [6]. With the rise of aesthetic awareness, "Pink Esthetics" has emerged, emphasizing restoration of soft tissue around teeth. Aesthetic demands necessitate intact papilla and symmetric gingival outline. However, sulcular incisions may pose challenges in mobilizing papilla, leading to tissue loss and subsequent shrinkage during healing. To address this, a flap technique preserving papilla instead of splitting it has been developed to maintain papilla height post-surgery [7].

## Papilla preservation approaches:

Preservation of interdental papilla can be approached through surgical and non-surgical methods. Non-surgical strategies involve addressing underlying causes such as periodontal disease, traumatic tooth brushing, malpositioning of teeth, or midline diastema. Treatment may include scaling and root planning, oral hygiene reinforcement, tooth brushing modifications, tooth repositioning, or orthodontic interventions to establish contact points. Surgical techniques aim to preserve maximum gingival tissue for complete coverage of regenerative materials in osseous defects. Various surgical approaches like Papilla preservation flap, Modified papilla preservation flap, Simplified papilla preservation flap, Single flap approach, Entire papilla preservation flap, Minimally Invasive Surgical Techniques (MIST), Modified - Minimally Invasive Surgical Techniques (M-MIST) and the "Whale's tail" technique are available for achieving optimal preservation of interdental papilla [8].

## Papilla preservation flap (PPF):

Genon and Bender (1984) introduced a technique for esthetic purposes, later detailed by Takei *et al.* (1985) as the Papilla Preservation Flap (PPF) [9]. This approach, inspired by Genon's work, aimed at optimal interproximal coverage and facilitated bone graft placement, preventing graft material exfoliation [10]. The technique involves sulcular incisions around each tooth and through lingual/palatal flaps, with semilunar incisions at interdental papillae to allow intact movement of facial flaps. Checchi *et al.* (1988) further modified PPF, advocating horizontal incisions over the opposite side of bone defects to protect the regenerated area from oral exposure [11].

## Modified papilla preservation flap (MPPF):

In 1995, Cortellini *et al.* introduced the Modified Papilla Preservation Flap (MPPF). This technique involves a primary intrasulcular incision between two adjacent teeth, followed by a horizontal incision at the base of the papilla on the buccal side [12]. A full thickness buccal flap is then raised to the level of the buccal alveolar crest, extending the incision to the palatal aspect. Another horizontal incision in the interproximal supracrestal connective tissue elevates the papilla towards the palate. A full thickness palatal flap is then elevated to expose the defect, with reduction of papilla thickness for flap advancement. Vertical releasing incisions may be added in neighboring interproximal spaces for further flap advancement if needed [10].

## Simplified papilla preservation flap (SPPF):

To address esthetic concerns in teeth with narrow interproximal zones, Cortellini *et al.* introduced the Simplified Papilla Preservation Flap technique in 1999 [5]. This method involves an oblique incision across the papilla associated with the defect, starting from the gingival margin at the buccal line angle of the affected tooth to the mid-interproximal portion under the adjacent tooth's contact point. The incision extends intrasulcularly to partially dissect the adjacent papillae, allowing elevation of a buccal flap with 2-3 mm exposure of alveolar bone. A buccolingual horizontal incision is then made at the base of the papilla, followed by intrasulcular incisions in the palatal aspects of neighboring teeth, extending into the interdental papilla, allowing elevation of a full-thickness palatal flap [13].

## Single flap approach:

Conventional access flap surgery, despite drawbacks like postsurgical recession and patient discomfort, remains the primary method for periodontal pocket reduction. The Single Flap Approach (SFA) was developed to address these issues and treat intraosseous defects. It offers advantages such as better wound healing, minimal surgical trauma and improved esthetics compared to conventional methods. Microsurgical instruments were used for precise incisions to minimize tissue trauma and surgeries were performed under aseptic conditions using local anesthesia [14]. Sulcular incisions were made along the gingival margin and a full-thickness mucoperiosteal flap was reflected on either the buccal or palatal/lingual side, depending on the defect location. After root planning and debridement, the flap was repositioned and secured with non-resorbable sutures [15].

## Entire papilla preservation flap:

A novel surgical approach introduced by Aslan, Buduneli and Cortellini in 2017, aims to treat deep intrabony defects with periodontal regeneration. This technique involves preserving the affected papilla with a tunnel-like undercut incision. By keeping the interdental papilla intact, the technique stabilizes the blood clot and improves wound healing. A short buccal vertical releasing incision may be necessary for adequate contact during debridement. The EPP technique may reduce the risk of wound failure, particularly in the early healing phase, thereby preventing exposure of regenerative materials and enhancing optimal clinical outcomes [16].

## Non-incised papillae surgical approach:

Proposed in 2018 by Moreno Rodriguez and Caffesse, the Non-Invasive Papilla Preservation Approach (NIPSA) involves a buccal horizontal incision placed as apically as possible from periodontal defects and marginal tissues. This allows rising a mucoperiosteal flap coronally, providing apical access to the defect while keeping marginal tissues intact, acting as a protective roof for interproximal defects and preventing papilla collapse. The incision is extended mesiodistally to expose cortical bone. NIPSA offers clinical advantages such as easier flap stabilization, preservation of blood supply in interdental areas, minimized postsurgical shrinkage and continuity of gingival vessels with the periodontal ligament and lingual blood supply. Compared to traditional extended flaps, NIPSA preserves blood supply more effectively [17].

## Minimally invasive surgical technique:

Harrel and Rees (1995) introduced this approach aiming for minimal wounds and flap reflection and gentle tissue handling [18]. Combining a papilla preservation flap with high-power magnification-assisted surgery, as proposed by Cortellini and Tonetti (2007), enhances wound stability and minimizes patient morbidity [19]. Defect-associated interdental papilla access is achieved using either the SPPF (Cortellini *et al.* 1999) or MPPT (Cortellini *et al.* 1995), depending on interdental space width. Intracrevicular incisions preserve gingival height and width and minimal flap elevation exposes the bone crest. Defects are debrided with mini-curettes and power-driven instruments, protecting flaps with periosteal elevators and saline irrigations. Biologically active agents are applied before flap repositioning. Suturing involves a single modified internal mattress suture for primary papilla closure. Surgical procedures are aided by operating microscopes or magnifying loupes at x4 to x 16 magnifications [20, 21].

## Modified minimally invasive surgical technique:

The Modified Minimally Invasive Surgical Technique (M-MIST), developed by Cortellini and Tonetti in 2007, enhances flap stability and space maintenance for regeneration. It involves a tiny interdental access with elevation of only a buccal triangular flap, leaving the papilla connected to the root with supracrestal fibers. Soft tissue in the defect is dissected and removed, followed by careful root debridement. Preservation of vessels aids interdental tissue healing. The technique supports interdental soft tissues through the "hanging" papilla, ensuring flap stability and blood clot stability. Minimal trauma and passive suturing promote primary wound closure, with additional sutures used if necessary [20].

## The "Whale's Tail" technique:

The purpose of this technique is to achieve primary closure for regenerating an interdental osseous defect between maxillary central incisors with an aberrant frenal attachment. Two semilunar incisions below the mucogingival line on the buccal surface were used for better flap margin approximation [20]. Soft tissue healing depends on various factors, including incision technique, flap design and patient compliance. Incisions away from the defect reduced flap dehiscence risk and sutures placed distant from the defect minimized bacterial colonization. Preserving papillae in flap design aided primary closure and vascularization. Only perimeter sutures were needed post-treatment, reducing the risk of suture-related complications [21].

## Modified vestibular incision sub periosteal tunnel access:

M-VISTA is utilized for treating intrabony defects in the esthetic area, employing a tunneling approach to minimize flap-related complications. Vertical incisions are made beyond the mucogingival line near the defects for access, with intrasulcular incisions on midfacial surfaces avoiding papillae. Subperiosteal tunnel elevation is performed, unlike VISTA which uses partial-thickness flaps. Full-thickness flaps are raised to place bone materials for intrabony defect treatment. Coronally anchored suturing advances the mucogingival complex, requiring a minimum 2mm keratinized gingival width for gingival health. The incision is vestibular and the flap is raised in a tunnel manner [22].

## Peri implant papilla:

In implant therapy, achieving both function and esthetics is crucial. However, preserving peri-implant papilla remains challenging. Loss of peri-implant papilla not only affects esthetics but also leads to issues like food impaction and speech difficulties. Unlike natural teeth, implants lack cementum and periodontal ligament, resulting in peri-implant mucosa resembling scar tissue with higher collagen proportions and fewer fibroblasts [23, 24].

## Description of the surgical technique:

After anesthesia, the bone defect is determined by probing. A semilunar incision is made at least 3mm apical to the defect, ensuring intact papillary tissue covers the graft area upon suturing. In narrow interdental spaces, papilla trimming may be necessary. Flaps are reflected and interdental tissue freed using instruments. Flaps are held and margins cleaned to remove epithelium and excess granulation tissue. Implant material is placed and a loose cross mattress suture prevents material dislodgement. Flaps are replaced over the graft, sutures tightened and a surgical dressing applied.

## Conclusion:

Maintaining esthetics is crucial in periodontal surgery, with papilla preservation key to successful outcomes. Evolving from traditional to minimally invasive techniques like MIST, papilla preservation flaps are tailored to interdental space width, promoting soft tissue healing and minimizing trauma. These methods, often combined with GTR and bone grafting, enhance both clinical outcomes and esthetic results, especially in the esthetic zone.
